# Mid- to long-term complications and revision rates of robotic-assisted unicompartmental knee arthroplasty: a systematic review and meta-analysis

**DOI:** 10.3389/fsurg.2025.1619644

**Published:** 2025-10-07

**Authors:** Xiaolin Chen, Bowen Wang, Jianming Huang, Zheyuan Huang, Weizong Weng, Danlei Huang, Desheng Xie, Ying Zhang

**Affiliations:** 1 Department of Orthopaedic Surgery, Xiamen University Affiliated Chenggong Hospital, Xiamen, Fujian, China; 2 Department of Orthopaedics, The Affiliated Hospital Southwest Medical University, Luzhou, Sichuan, China

**Keywords:** unicompartmental knee arthroplasty, osteoarthritis, complication, revision rate, meta-analysis

## Abstract

**Objective:**

Unicompartmental knee arthroplasty (UKA) is a commonly performed procedure for patients with isolated osteoarthritis (OA). In recent years, robotic-assisted UKA (RAUKA) has raised concerns regarding its revision rates and risk of complications. This study aims to compare the mid- to long-term complications and revision rates between RAUKA and traditional UKA, providing evidence to support its clinical application.

**Methods:**

In accordance with PRISMA guidelines, we conducted a systematic review of studies comparing complication and revision rates between RAUKA and traditional UKA, with a minimum average follow-up duration of three years. Comprehensive searches were conducted in PubMed, Embase, Web of Science, and Cochrane databases, with a cutoff date of October 1, 2024. The outcome measures analyzed included complications, revision rate, postoperative aseptic loosening, fractures, malalignment, pain, and OA.

**Results:**

Six studies were included, encompassing 48,091 knee cases, with follow-up durations ranging from 36–106.4 months. RAUKA significantly reduced the overall complication rate (odds ratio: 0.27, 95% CI: 0.11–0.63, *P* = 0.003) and revision rate (odds ratio: 0.28, 95% CI: 0.12–0.67, *P* = 0.004) compared to traditional UKA. RAUKA also significantly reduced the incidence of postoperative aseptic loosening (odds ratio: 0.29, 95% CI: 0.17–0.50, *P* < 0.001) and fractures (odds ratio: 0.20, 95% CI: 0.05–0.79, *P* = 0.020). However, no significant differences were found between the two groups for postoperative malalignment, pain, or secondary OA.

**Conclusions:**

This study is the first to include mid- to long-term follow-up (≥3 years) data comparing RAUKA and traditional UKA. The findings indicate that RAUKA outperforms traditional UKA in terms of overall complication and revision rates, with a lower incidence of key complications such as postoperative aseptic loosening and fractures. RAUKA appears to be a safer surgical option for OA patients, supporting its broader clinical application. However, further long-term, multicenter studies are needed to fully validate its efficacy and long-term safety.

**Systematic Review Registration:**

identifier [ID CRD42024605539].

## Introduction

Unicompartmental knee arthroplasty (UKA) is a cost-effective surgical approach, commonly used to reliably treat OA confined to the medial or lateral compartment of the knee, while preserving ligaments and bone (1, 2). In recent years, the number of UKA procedures has been steadily increasing (3). Reports indicate that, compared to total knee arthroplasty (TKA), UKA has a lower complication rate, higher patient satisfaction, and faster recovery (4, 5). However, the drawbacks of UKA include some postoperative complications and a relatively high failure rate. Early aseptic loosening and malalignment, as the most common complications of UKA, are considered major reasons for UKA surgical failure (6, 7).

In recent years, with the development and clinical application of various robotic-assisted systems, joint replacement surgeries have become more precise. Statistics indicate that approximately 20% of UKA procedures in the United States are performed with robotic assistance, and this proportion is continuing to rise (8, 9). Robotic-assisted UKA (RAUKA) holds promises for improving clinical outcomes by enhancing surgical precision and positioning accuracy (10). However, there are still conflicting views on the impact of RAUKA on complication rates and revision rates (11, 12). For instance, a meta-analysis reported that while RAUKA demonstrated better early functional outcomes, no evidence shows advantage in revision rate (13). On the other hand, Zhang et al. (14) found that RAUKA significantly reduced complication rates and improved knee alignment. Thus, further evidence and research are needed to clarify the effects of RAUKA on patient complications and revision rates.

A recent study investigated the differences between RAUKA and conventional UKA regarding complications and revision rates. Apart from knee function, there were no significant differences in other outcomes (15). However, the follow-up duration of the included studies ranged from 1–46 months, which may influence the comparison of data on complications and revision rates. As an emerging and promising technology, RAUKA requires attention to its long-term complications and revision rates. Current studies and analyses mainly focus on early complication risks and do not discuss key complications such as aseptic loosening and malalignment. Therefore, this study incorporated various types of research with an average follow-up time of ≥3 years to explore the differences in mid- to long-term complications and revision rates between RAUKA and conventional UKA, providing theoretical support for the clinical application and long-term efficacy of RAUKA.

## Materials and methods

This systematic review and meta-analysis adhered to the guidelines outlined by the Preferred Reporting Items for Systematic Reviews and Meta-Analyses (PRISMA) (16). The study was registered with the International Prospective Register of Systematic Reviews (PROSPERO) under the ID CRD42024605539 prior to initiating the database search and study selection process.

## Search strategy

We conducted a literature search in the PubMed, Embase, Web of Science, and Cochrane databases, covering publications from the inception of the databases to October 1, 2024. The following combinations of keywords were used: (robotic-assisted unicompartmental knee arthroplasty OR traditional unicompartmental knee arthroplasty OR conventional unicompartmental knee arthroplasty OR manual unicompartmental knee arthroplasty OR unicompartmental knee replacement OR partial knee arthroplasty OR partial knee replacement) AND (complications OR revision OR effect OR outcome OR efficacy). Two authors independently screened the retrieved literature and further evaluated based on inclusion criteria from titles and/or abstracts. Any disagreements were resolved through discussion with a third senior author. Full-text articles that met the inclusion criteria were thoroughly reviewed, and their references were manually checked to ensure that all relevant studies were included. Additionally, overlapping and duplicate data were identified and excluded.

## Inclusion and exclusion criteria

The literature was included in the study if it met the following criteria:

1. Clinical research evidence at any level [including randomized controlled trials (RCTs), case-control studies, prospective cohort studies, and retrospective comparative studies];
2. Studies published in English;
3. Reporting data on the number of cases with complications;
4. Studies with a minimum average follow-up of three years postoperatively.

The exclusion criteria are as follows:

1. Reviews, abstracts, letters, commentaries, case reports, and non-case-control studies;
2. Preclinical studies based on cell, animal models, or cadaveric research;
3. Studies reporting fewer than 10 cases in the experimental or control group (to ensure the analysis of the outcomes of interest and the reliability of results);
4. Studies that do not report complication and revision data.

## Data extraction and quality assessment

Two independent authors inspected and extracted data from the included literature, which was then placed into a pre-created Microsoft Excel sheet and saved. The specific data extracted included the following: the first author's surname, year, country, study design type, age, gender, number of patients (number of knees), follow-up time, revision rate due to any cause, number of complications, details of complications, and the robotic system used. The Newcastle-Ottawa Scale (NOS) was used to assess the quality of the included studies (17). Specifically, two independent and experienced authors rated the studies, and the final score was determined through discussion and consolidation with a senior third author. A score of 9 indicated a high-quality study, scores of 6–8 indicated good-quality studies, scores of 3–5 indicated moderate-quality studies, and a score of less than 3 indicated a low-quality study.

## Outcomes of interest

In this study, we assessed several outcomes of interest, including two primary outcomes and five secondary outcomes. The primary outcomes included overall revision rate and complication incidence. Overall revision rate was defined as the proportion of patients undergoing any surgical procedure involving removal or exchange of any component of the UKA implant, regardless of the reason. Overall complication incidence was defined as the proportion of patients experiencing any adverse event related to the surgery or implant requiring intervention. The secondary outcomes included postoperative aseptic loosening, fractures, malalignment, pain, and progression of osteoarthritis. Each above outcome indicator was reported as a pooled incidence rate.

## Statistical analysis

The differences between robotic-assisted and conventional UKA for binary variables (complication incidence and revision rate) were analyzed by calculating the odds ratio, with a 95% confidence interval (CI). Heterogeneity was assessed using the *χ*² test and the *I*² statistic (18). According to Cochrane Handbook standards, 0%–40% may represent low heterogeneity, 30%–60% may represent moderate heterogeneity, 50%–90% indicates substantial heterogeneity, and 75%–100% suggests considerable heterogeneity. When *I*² ≤ 50% and *P* > 0.10, a fixed-effect model was used; Otherwise, a random-effects model was applied for the pooled effect analysis. All data were analyzed using RevMan version 5.3 software (The Cochrane Collaboration, Copenhagen, Denmark). A *P*-value of <0.05 was considered statistically significant.

## Results

### Literature screening process

**Figure 1:**
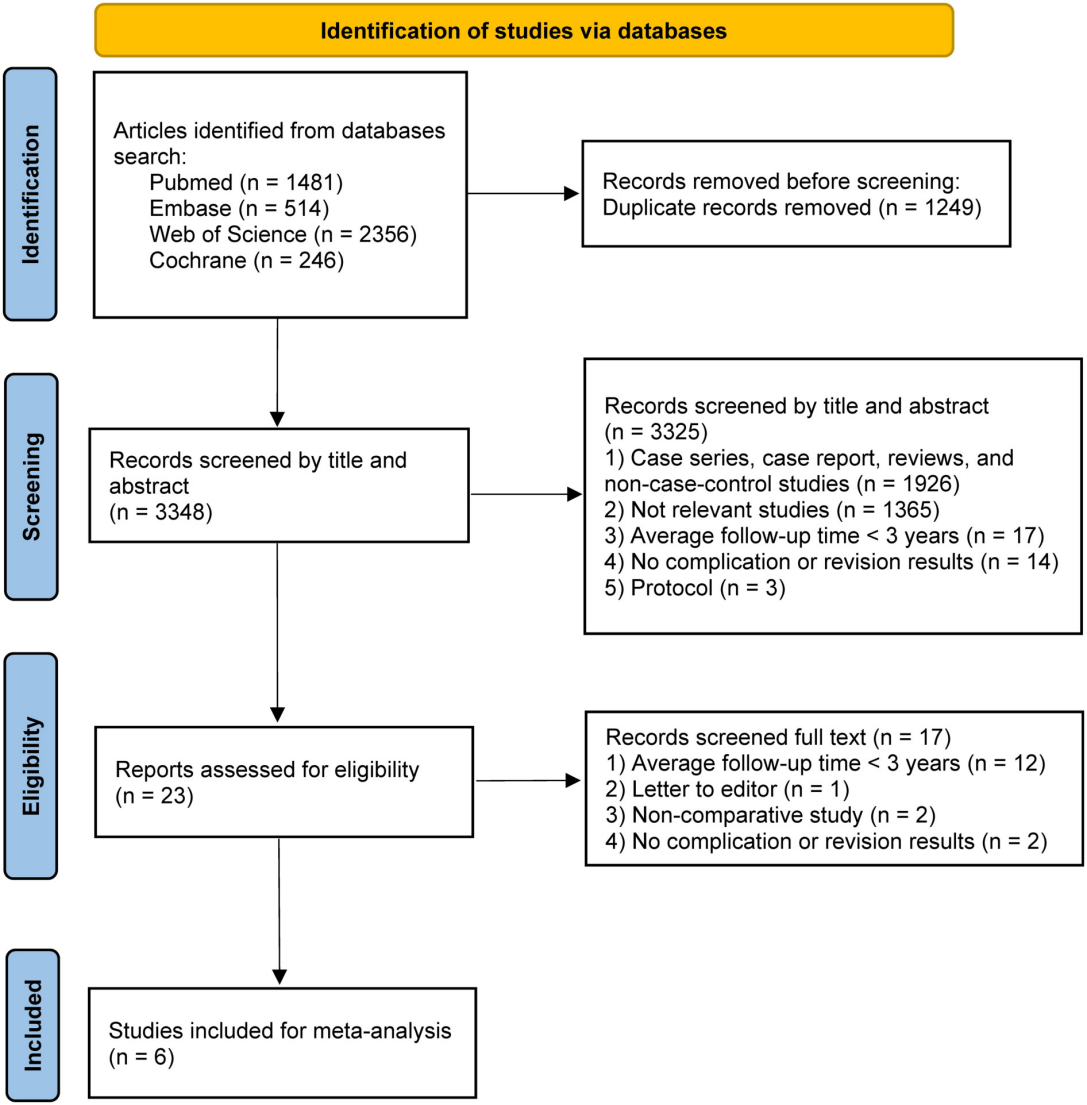
Flowchart of the study based on the PRISMA guidelines.

Through an initial search of the databases, 1,929 relevant articles were identified. First, 403 duplicate articles were manually removed. Next, further screening was performed based on titles and abstracts, excluding 1,503 ineligible studies, including those with irrelevant topics, inappropriate article types, or non-English publications. Finally, after downloading and further screening the full texts, studies with an average follow-up period of less than 3 years and those lacking descriptions of complications were excluded. In the end, 6 studies (19–24) met our comprehensive inclusion criteria and were selected for further analysis. The PRISMA flow diagram for this study is shown in Figure 1.

### Characteristics of included studies

The six rigorously selected studies were conducted between 2018 and 2023, with each study representing one year. Specifically, a total of 48,091 knee cases were analyzed, of which 16,793 underwent RAUKA, while the remaining 31,298 received conventional UKA. The overall patient age ranged from 49.6–76.4 years, with follow-up periods ranging from 36–106.4 months. Regarding the robotic-assisted systems used during surgery, one study did not specify the system, three studies utilized the MAKO system, and two studies used the Navio system. In terms of study design, one study was a randomized controlled trial (RCT), while the rest were retrospective cohort or retrospective comparative studies. All studies reported postoperative complications and revision rates. Table 1 summarizes the basic characteristics of the six included studies.

**Table 1:** Main characteristics of all articles included in the meta-analysis.

Author	Year	Country	Study design	Robot systems	Implants	Age (RAUKA vs. UKA, Years ± SD, range)	Number of female/male (RAUKA vs. UKA)	Number of knees (RAUKA vs. UKA)	Follow-up time (RAUKA vs. UKA, month)	Revision rates	Complication cases	Detail of complications
Canetti et al (19)	2018	France	Retrospective cohort	Navio system (Smith and Nephew)	A cemented resurfacing unicompartmental prosthesis with an all-polyethylene tibial component (HLS Uni Evolution, Tornier®)	66.5 ± 6.8 vs. 59.5 ± 9.9	9/2 vs. 12/5	11 vs. 17	34.4 ± 10.5 vs. 39.3 ± 15.5	0 vs. 5.88%	0 vs. 1	UKA 1 case (common peroneal nerve paralysis after surgery)
Banger et al (20)	2019	UK	RCT	RIO System (MAKO)	No description	No description	No description	65 vs. 74	Average 60	0 vs. 2.7%	0 vs. 6	RAUKA 0 case; UKA 6 cases (2 revisions, 1 poly exchange and 3 arthroscopies)
St Mart et al (21)	2020	Australia	Retrospective comparative study	Mako-assisted Restoris (MAKO)	An onlay polyeth ylene insert	Average 66 vs. 65	1,243/1,608 vs. 4,078/5,483	2,851 vs. 9,561	Average 46	1.65 vs. 3.15%	47 vs. 301	RAUKA 47 cases (18 infections, 10 loosening, 8 progression of disease, 4 pain, 3 instability, 1 fracture, 1 malalignment and 2 other); UKA 301 cases (25 infections, 114 loosening, 64 progression of disease, 18 pain, 8 instability, 26 fracture, 8 malalignment and 36 other)
Vakharia et al	2021	USA	Retrospective comparative study	No description	No description	No description	6,753/5,794 vs. 10,818/10,209	13,617 vs. 21,444	Average 36	0.918 vs. 6.19%	125 vs. 1,327	No description
Maritan et al (23)	2022	Italy	Retrospective cohort	MAKO robotic arm system (Stryker)	No description	60.9 ± 8.4 vs. 61.5 ± 8.5	41/11 vs. 37/6	52 vs. 43	95.4 ± 11.0 vs. 90.3 ± 9.1	3.85 vs. 6.98%	2 vs. 3	RAUKA 2 cases (1 patellofemoral osteoarthritis and 1 inexplicable pain); UKA 3 case (2 aseptic loosening and 1 periprosthetic fracture)
Foissey et al (24)	2023	France	Retrospective cohort	BlueBelt Navio robotic surgical system	HLS Uni Evolution, Tornier®	66.7 ± 7.7 vs. 68.3 ± 8.1	108/89 vs. 98/61	197 vs. 159	61.3 ± 24.0	3.05 vs. 12.58%	6 vs. 20	RAUKA 6 cases (5 tibial aseptic loosening and 1 tibial plateau fracture); UKA 20 case (18 tibial aseptic and 2 femoral loosening, 2 lateral OA, 2 malalignment, 1 tibial plateau fracture)

RAUKA, robotic-assisted unicompartmental knee arthroplasty; UKA, unicompartmental knee arthroplasty.

### Quality assessment

After meticulous scoring by two independent authors using the NOS and final aggregation, all studies were assessed as moderate to high quality (Table 2). Specifically, four studies received scores of 4–6, categorizing them as moderate quality, while two studies scored 7, classifying them as high quality.

**Table 2:** The results of quality assessment for included studies using the Newcastle-Ottawa scale.

Study	Selection				Comparability	Exposure			Scores
	Adequate definition of cases	Representativeness of the cases	Selection of controls	Definition of controls	Control for important factors^a^	Ascertainment of exposure	Same method of ascertainment for cases and controls	Non-response rate	
Canetti et al. 2018	★		★			★	★	★	5
Banger et al. 2019	★	★	★		★	★	★	★	7
St Mart et al. 2020	★	★	★		★	★	★	★	7
Vakharia et al. 2021	★		★				★	★	4
Maritan et al. 2022	★		★		★	★	★	★	6
Foissey et al. 2023	★		★		★	★	★	★	6

a A maximum of 2 stars can be allotted in each category, one for age, and the other for other controlled factors.

## Clinical outcomes

### Revision rate

**Figure 2:**
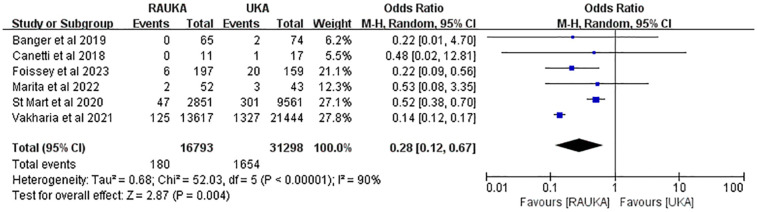
Forest plot shows the difference in overall revision rate between RAUKA and conventional UKA. Odds ratio (OR), 95% confidence interval (95% CI).

Revision rate is a key indicator of postoperative efficacy, and all six studies reported knee revisions over the average follow-up period. We analyzed the difference in medium- to long-term revision rates between RAUKA and UKA. The *χ*² and *I*² tests (*P* < 0.00001, *I*² = 90%) indicated substantial heterogeneity, so a random-effects model was applied. The analysis showed that the medium- to long-term revision rate for RAUKA was significantly lower than that for conventional UKA (odds ratio: 0.28, 95% CI: 0.12–0.67, *P* = 0.004) (Figure 2).

### Complications

**Figure 3:**
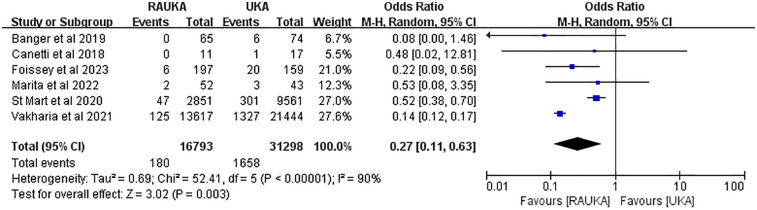
Forest plot shows the difference in overall complication rates between RAUKA and conventional UKA. Odds ratio (OR), 95% confidence interval (95% CI).

To investigate whether there is a significant difference in complications between RAUKA and conventional UKA in medium- to long-term follow-up (≥3 years), we collected all complication data. Overall, the reported complications included aseptic loosening, fracture, infection, pain, instability, malalignment, prosthesis dislocation, lateral OA, postoperative common peroneal nerve paralysis, and other adverse events. All studies reported the number of complication cases in both the RAUKA and UKA groups. The *χ*² and *I*² tests yielded *P* < 0.00001 and *I*² > 90%, indicating statistical heterogeneity among studies, so a random-effects model was used for pooled effect analysis. The complication analysis results showed that, with an average follow-up of at least 3 years, the complication rate for RAUKA was significantly lower than that for conventional UKA (odds ratio: 0.27, 95% CI: 0.11–0.63, *P* = 0.003) (Figure 3).

**Figure 4:**
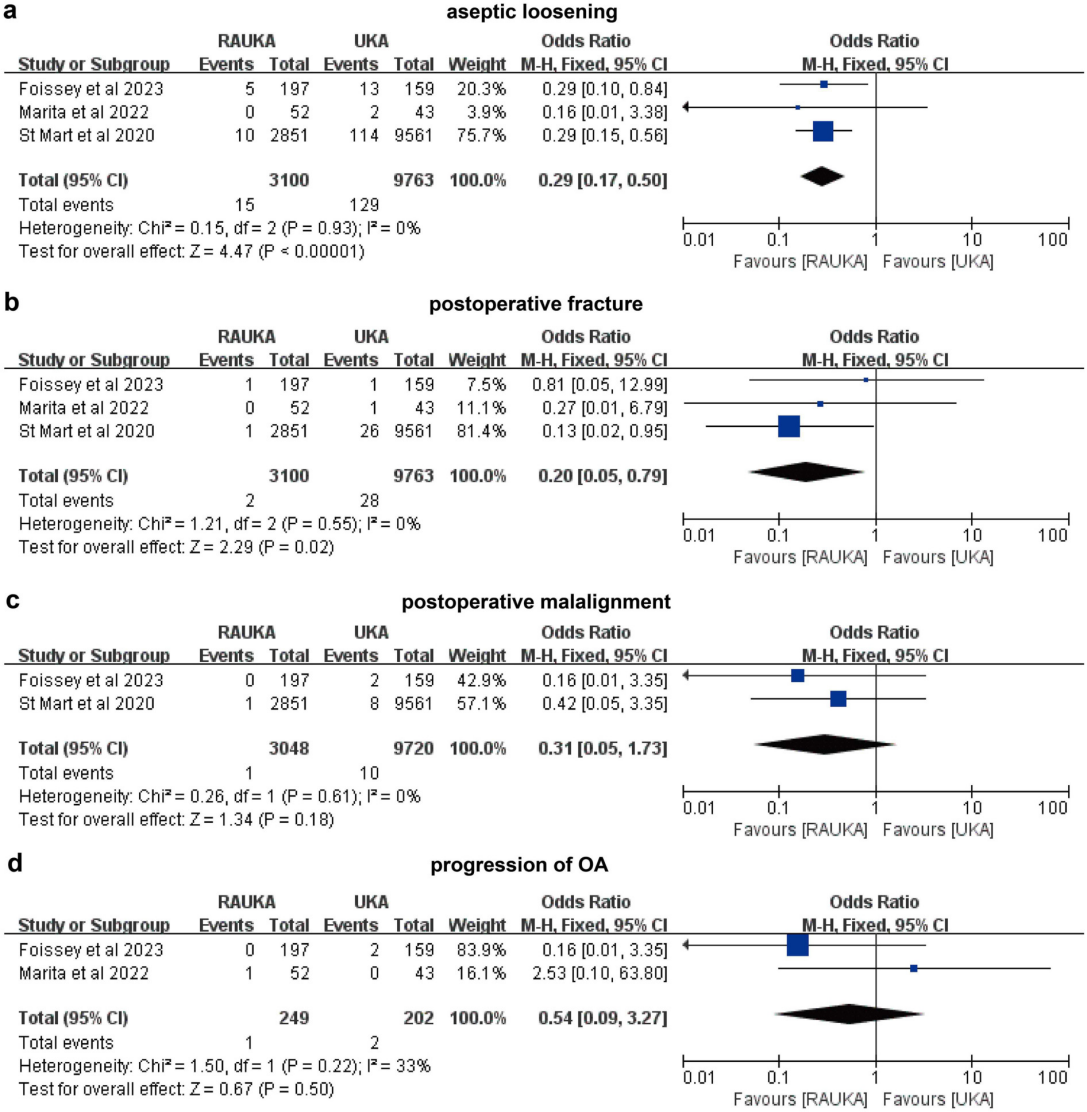
Forest plots illustrate the differences in incidence rates of various complications between RAUKA and conventional UKA. **(a)** postoperative aseptic loosening; **(b)** postoperative fracture; **(c)** postoperative malalignment; **(d)** postoperative OA. Odds ratio (OR), 95% confidence interval (95% CI).

Additionally, we further analyzed the incidence of specific complications. Specifically, three studies reported on aseptic loosening, three on postoperative fractures, two on malalignment, two on postoperative pain, and two on postoperative OA. In the analysis related to aseptic loosening, using a fixed-effects model (*P* = 0.93, *I*² = 0%), the pooled results showed that the incidence of postoperative aseptic loosening was significantly lower in RAUKA compared to conventional UKA (odds ratio: 0.29, 95% CI: 0.17–0.50, *P* < 0.001) (Figure 4a). In the analysis of postoperative fractures, also using a fixed-effects model (*P* = 0.55, *I*² = 0%), the pooled results indicated that the incidence of postoperative fractures was significantly lower in RAUKA than in conventional UKA (odds ratio: 0.20, 95% CI: 0.05–0.79, *P* = 0.020) (Figure 4b).

For the incidence of malalignment, we conducted a pooled analysis using a fixed-effects model (*P* = 0.61, *I*² = 0%), which showed no significant difference in postoperative malalignment rates between RAUKA and conventional UKA (odds ratio: 0.31, 95% CI: 0.05–1.73, *P* = 0.180) (Figure 4c). In the analysis of postoperative pain incidence, the fixed-effects model (*P* = 0.48, *I*² = 0%) also indicated no significant difference between RAUKA and conventional UKA (odds ratio: 0.85, 95% CI: 0.32–2.30, *P* = 0.750) (Figure 3d). Lastly, two studies reported on the incidence of progression of OA after surgery, and the fixed-effects model analysis (*P* = 0.22, *I*² = 33%) also suggested no significant difference between the two groups (odds ratio: 0.54, 95% CI: 0.09–3.27, *P* = 0.500) (Figure 4d).

## Discussion

The most important findings were that RAUKA significantly reduced the overall complication rate (odds ratio: 0.27, 95% CI: 0.11–0.63, *P* = 0.003) and revision rate (odds ratio: 0.28, 95% CI: 0.12–0.67, *P* = 0.004) compared to conventional UKA in mid- to long-term follow-up (≥3 years). Additionally, RAUKA demonstrated a significantly lower incidence of postoperative aseptic loosening (odds ratio: 0.29, 95% CI: 0.17–0.50, *P* < 0.001) and fractures (odds ratio: 0.20, 95% CI: 0.05–0.79, *P* = 0.020). However, no significant differences were observed between the two groups in terms of postoperative malalignment, pain, or secondary osteoarthritis. This focus on ≥3-year outcomes fills an important gap in the literature, as most previous reviews have centered on early complications, while the mid- to long-term safety profile of RAUKA has remained underexplored.

Compared to TKA, UKA offers several advantages and is an effective treatment for unicompartmental knee OA (25–27). However, complications and revision rates have consistently been major concerns for orthopedic surgeons. A 27-year Finnish registry study found that the survival rate for UKA was significantly lower than that for TKA (28). The 5-year survival rate for UKA was 89.4%, the 10-year rate was 80.6%, and the 15-year rate was 69.6%, while TKA survival rates at the same follow-up intervals were 96.3%, 93.3%, and 88.7%, respectively. Ma et al. (29) compared the mid-term follow-up results of simultaneous UKA on one knee and TKA on the other in the same patient, finding similar complication rates but a higher prosthetic revision rate for UKA. Additionally, a meta-analysis by Evans et al. (30) reported an estimated 25-year survival rate of 72% for UKA, compared to 82.3% for TKA. Therefore, surgical complications and revision rates remain critical issues that must be addressed for wider clinical adoption of UKA.

In recent years, robotic-assisted systems have attracted significant interest due to their potential to reduce complications and revision rates in UKA (31). These systems enhance component positioning and dynamic ligament balancing, thereby improving clinical outcomes (32, 33). As a result, studies investigating the clinical efficacy, complications, and revision rates of RAUKA have been ongoing. Regarding primary outcomes, a meta-analysis by Sun et al. (15) indicated that RAUKA results in fewer complications and a lower revision rate. However, a single-center case-control study suggested that while RAUKA showed a lower revision rate in short-term follow-up compared to traditional UKA, there was no significant difference in the incidence of complications (34). An earlier meta-analysis by Zhang et al. (14) found that RAUKA significantly reduced complication risk but did not show a significant difference in revision rates between the two groups. Another study reported that although RAUKA significantly improved short-term functional outcomes, there was no significant difference in revision rates or medium- to long-term functional outcomes when compared to traditional UKA (13). To assess the impact of RAUKA on mid- to long-term complications and revision rates, our pooled analysis of studies with a mean follow-up duration of ≥3 years revealed that RAUKA significantly reduced both complication and revision rates compared to traditional UKA. However, further long-term follow-up data and additional evidence are needed for confirmation.

Previous studies have primarily focused on overall knee function and the total incidence of complications, with limited attention given to the specific incidence rates of individual complications (35, 36). In our study, we further analyzed five complications: postoperative aseptic loosening, fractures, malalignment, pain, and OA. Previous research has shown that aseptic loosening, particularly tibial component loosening, is a leading cause of revision in UKA and may increase the risk of postoperative fractures (37, 38). The introduction of robotic-assisted systems could potentially reduce the incidence of these complications. Our findings support this, as we observed significantly lower rates of postoperative aseptic loosening and fractures in RAUKA compared to traditional UKA. Furthermore, in younger patients, revision surgery was significantly associated with aseptic loosening and pain (39). However, despite the significantly lower postoperative revision rate in RAUKA, there were no statistically significant differences between the two groups regarding the incidence of postoperative pain, malalignment, or OA. Ghazal et al. (40) included 12 studies comparing RAUKA and traditional UKA in terms of knee function and outcomes. Their results indicated no significant differences between the two methods for certain complications, including pain, which is consistent with our findings.

It is noteworthy that, compared to traditional UKA, RAUKA typically involves longer surgical times, which may increase the risk of infection-related complications (41, 42). A follow-up analysis of 11,633 UKA procedures in the United States examined the impact of surgical duration on short-term complications, including surgical site infections, reoperation rates, and mortality (43). The results revealed a significant association between longer operative times and an increased risk of short-term postoperative complications. However, since only one study in our analysis reported infection-related complications, we were unable to perform a meta-analysis to compare the differences between RAUKA and conventional UKA.

Our meta-analysis has several limitations. Firstly, while our study aimed to explore the differences in mid- to long-term complications and revision rates between RAUKA and traditional UKA, the limited average follow-up duration posed a constraint. After further filtering the 23 comparative studies on RAUKA and UKA based on an average follow-up time of ≥3 years, only six studies met this criterion, preventing us from conducting more detailed mid- and long-term subgroup analyses. Secondly, two of the studies included did not provide detailed information on complications; although we attempted to contact the authors, we received no response. Consequently, only two or three studies were included in the specific complication analysis, necessitating cautious interpretation of these results. Thirdly, although our study aimed to explore differences in medium- to long-term (≥3 years) complications and revision rates, only one included study had a follow-up period exceeding 10 years. Therefore, no definitive conclusions can be drawn regarding the long-term survival of RAUKA implants, and high-quality studies with longer follow-up durations are still needed. Furthermore, due to limitations in patient numbers, heterogeneity across studies, and variability in study quality, the conclusion that RAUKA has a lower mid- to long-term complication and revision rate requires further validation through multicenter, prospective, and randomized controlled trials.

## Conclusions

For OA patients undergoing joint replacement surgery, RAUKA demonstrated a lower overall complication and revision rate in the mid- to long-term (with an average follow-up of at least three years) compared to traditional UKA. Notably, RAUKA also showed a reduced incidence of common complications associated with tibial component loosening, such as postoperative aseptic loosening and fractures. These findings provide valuable insight into the long-term efficacy and potential for broader clinical adoption of RAUKA. However, further long-term follow-up studies are needed to confirm its safety and clinical effectiveness.

## Data Availability

The raw data supporting the conclusions of this article will be made available by the authors, without undue reservation.
