# Supplementary material for: Mid- to long-term complications and revision rates of robotic-assisted unicompartmental knee arthroplasty: a systematic review and meta-analysis
Source: Front Surg. 2025 Oct 7;12:1619644. doi: 10.3389/fsurg.2025.1619644 (PMC12537783; doi:10.3389/fsurg.2025.1619644)
Supplement: Supplementary file 2 [file Datasheet2.docx]

**Supplement** [**Materials**](D:/%E8%BD%AF%E4%BB%B6/360%E6%B8%B8%E8%A7%88%E5%99%A8/Youdao/Dict/8.9.9.0/resultui/html/index.html#/javascript:;)

**The Detailed Search Strategy:**

**Pubmed Cut-off retrieval date: October 1, 2024**

#1: “robotic-assisted unicompartmental knee arthroplasty” OR “traditional unicompartmental knee arthroplasty” OR “conventional unicompartmental knee arthroplasty” OR “manual unicompartmental knee arthroplasty” OR “unicompartmental knee replacement” OR “partial knee arthroplasty” OR “partial knee replacement”

Results: 1,792

#2: “complications” OR “revision” OR “effect” OR “outcome” OR “efficacy”

Results: 10,051,647

#3: #1 AND #2

**Results: 1,481**

**Cochrane Library Cut-off retrieval date: October 1, 2024**

#4: (robotic-assisted unicompartmental knee arthroplasty):ab,ti,kw OR (traditional unicompartmental knee arthroplasty):ab,ti,kw OR (conventional unicompartmental knee arthroplasty):ab,ti,kw OR (manual unicompartmental knee arthroplasty):ab,ti,kw OR (unicompartmental knee replacement):ab,ti,kw OR (partial knee arthroplasty):ab,ti,kw OR (partial knee replacement):ab,ti,kw

Results: 359

#5: (complications):ab,ti,kw OR (revision):ab,ti,kw (effect):ab,ti,kw OR (outcome):ab,ti,kw OR (efficacy):ab,ti,kw

Results: 1,043,596

#6: #4 OR #5

**Results: 246**

**Web of Science Cut-off retrieval date: October 1, 2024**

#7: TS=(robotic-assisted unicompartmental knee arthroplasty OR traditional unicompartmental knee arthroplasty OR conventional unicompartmental knee arthroplasty OR manual unicompartmental knee arthroplasty OR unicompartmental knee replacement OR partial knee arthroplasty OR partial knee replacement)

Results: 3,384

#8: TS=(complications OR revision OR effect OR outcome OR efficacy)

Results: 13,994,562

#9: #7 AND #8

**Results: 2,356**

**Embase Cut-off retrieval date: October 1, 2024**

#10: 'robotic-assisted unicompartmental knee arthroplasty':ab,ti,kw OR 'traditional unicompartmental knee arthroplasty':ab,ti,kw OR 'conventional unicompartmental knee arthroplasty':ab,ti,kw OR 'manual unicompartmental knee arthroplasty':ab,ti,kw OR 'unicompartmental knee replacement':ab,ti,kw OR 'partial knee arthroplasty':ab,ti,kw OR 'partial knee replacement':ab,ti,kw

Results: 874

#11: 'complications':ab,ti,kw OR 'revision':ab,ti,kw OR 'effect':ab,ti,kw OR 'outcome':ab,ti,kw OR 'efficacy':ab,ti,kw

Results: 9,966,236

#12: #10 AND #11

**Results: 514**
